# A Hybrid Sequencing Approach Completes the Genome Sequence of Thermoanaerobacter ethanolicus JW 200

**DOI:** 10.1128/MRA.01530-18

**Published:** 2019-01-17

**Authors:** Richard K. Tennant, Monica L. Ayine, Ann L. Power, James A. Gilman, Mark Hewlett, Paul B. C. James, Chloe Singleton, David A. Parker, John Love

**Affiliations:** a Biosciences, College of Environmental and Life Sciences, The BioEconomy Centre, University of Exeter, Exeter, United Kingdom; University of Rochester School of Medicine and Dentistry

## Abstract

Thermoanaerobacter ethanolicus JW 200 has been identified as a potential sustainable biofuel producer due to its ability to readily ferment carbohydrates to ethanol. A hybrid sequencing approach, combining Oxford Nanopore and Illumina DNA sequence reads, was applied to produce a single contiguous genome sequence of 2,911,280 bp.

## ANNOUNCEMENT

Thermoanaerobacter ethanolicus JW 200 is a thermophilic Gram-positive obligate anaerobe originally isolated from Yellowstone National Park (1). T. ethanolicus JW 200 readily ferments glucose to ethanol and has received attention as a sustainable biofuel producer (2) and for its bioconversion capabilities (3).

T. ethanolicus JW 200 was obtained from the DSMZ (DSMZ 2246) and was cultured in Thermoanaerobacter medium (DSMZ 61) at 65°C. DNA was purified in an anaerobic cabinet using a combination of the MP Bio FastDNA spin kit and a high-molecular-weight genomic DNA (gDNA) extraction protocol (4). An overnight culture was resuspended in CLS-TC buffer (MP Bio, USA) and transferred to a tube containing lysing matrix A (MP Bio, USA). Lysis tubes were placed into MP Bio FastPrep and homogenized at 6.0 m·s^−1^ for 40 s. Centrifuged lysates were treated with RNase A, and genomic DNA was isolated using AMPure XP beads. Purified DNA was quantified using a Qubit fluorometer (Thermo Fisher Scientific, UK), and DNA quality was measured using the genomic TapeStation assay tapes (Agilent Technologies, USA). Illumina DNA sequencing libraries were prepared with the Nextera XT library prep kit (Illumina, USA) and sequenced by an Illumina MiSeq instrument, using 150-bp paired-end sequencing. Oxford Nanopore libraries were prepared using the SQK-LSK108 1D genomic DNA ligation kit (Oxford Nanopore, UK) and sequenced on a MinION instrument using an R9.4a flow cell (Oxford Nanopore). Oxford Nanopore data were base called using Albacore v2.3.3, and DNA sequence data were verified using Porechop (5), yielding 11.37 Gbp of data, with an *N*_50_ value of 6,401 bp. Illumina DNA sequence data were quality controlled and filtered using TrimGalore v0.3.3 (6) with the “paired” parameter selected, which yielded 2,031,855 paired-end reads with an *N*_90_ value of 138 bp. A hybrid assembly of the Oxford Nanopore and Illumina data was performed by MaSuRCA v3.2.3 (7) using the default parameters. The genome was assembled to a single 2,911,280-bp contiguous sequence with a GC content of 34.2% and more than 3,500-fold genome coverage. The assembled genome was verified by aligning the reads against the *de novo* assembled genome using BWA-MEM v0.7.15 (8) with the default parameters. The alignment was visualized in Tablet v1.17 (9) to ensure complete coverage. The completed T. ethanolicus genome was annotated using Prokka v1.12 (10), and 2,818 coding sequences (CDS) were identified.

### Data availability.

The complete genome sequence of T. ethanolicus JW 200 is deposited in GenBank under the accession number CP033580. Illumina and Oxford Nanopore DNA sequence reads have been deposited in the NCBI Sequence Read Archive (accession numbers SRR8113455 and SRR8113456).
